# Supplementary material for: Are carbon emissions trading and green financial instruments synergistic? -Comprehensive quantitative research based on content analysis
Source: PLoS One. 2024 Mar 7;19(3):e0298601. doi: 10.1371/journal.pone.0298601 (PMC10919601; doi:10.1371/journal.pone.0298601)
Supplement: S1 Data — (ZIP) [file pone.0298601.s001.zip › Data/地级市Z_F工作报告.zip!/─┌├╔╣┼/║⌠┬╫▒┤╢√╩╨/└┤╫╘╣1⁄2╓┌║┼í╛┬φ┐╦╩2╛▌═°í┐_2009.docx]

各位代表：

现在，我代表呼伦贝尔市人民政府向大会作工作报告，请予审议，并请市政协委员和列席会议的同志们提出意见。

一、2008年工作回顾

2008年是很不寻常的一年。在自治区党委、政府和市委的正确领导下，我们紧紧依靠全市各族人民，深入贯彻落实科学发展观，沉着应对复杂多变的国内外经济形势，成功战胜了春旱、火灾等自然灾害，有效破解了发展中的各种难题，实现了经济社会又好又快发展。

——经济实力稳步增强。地区生产总值跨过600亿元大关，完成632.66亿元，增长15％。财政总收入历史性突破100亿元，完成100.12亿元，增长42.6％；地方财政总收入完成74.21亿元，增长29.1%。实施超亿元工业项目59个，其中超10亿元项目19个；规模以上工业增加值完成164.5亿元，增长22%。农牧业喜获丰收，粮食总产量达到90亿斤，增产20亿斤；牧业年度牲畜存栏1447万头只，农牧业产业化龙头企业发展到124家，农畜产品加工率达到60%以上；林区经济实力进一步提升，我市跻身国家现代林业示范市行列。社会消费品零售总额完成190亿元，增长24.9％。旅游收入完成93亿元，增长33%；接待游客611万人次，增长27%。城镇居民人均可支配收入完成12099元，增长16.75％；农牧民人均纯收入完成5061元，增长20.19％。城镇登记失业率完成4.05%，控制在自治区下达的4.3%以内。全面完成了自治区下达的节能减排目标任务。

——改革开放深入推进。企业改革逐步深化，森工集团企业剥离办社会职能改革和主辅分离扎实推进，首批移交地方12477人。粮食流通体制改革进展顺利，全面完成了国债和世行项目库上划工作。财税体制改革成效明显，持续推进了部门预算和国库集中支付改革。全面完成了交通养路费管理体制上划工作。非公有制经济进一步发展壮大，占国民经济总量的41％。口岸基础设施日趋完善，完成了满洲里公路口岸续建、海拉尔航空口岸机场国际候机楼等基础设施建设，全市口岸通关能力提升到4000万吨和300万人次。克服金融危机的冲击，外贸进出口总额完成27.8亿美元，增长3%；进出口货运量达到2434万吨，增长0.4%。引进国内（市外）资金338.55亿元，增长33%。

——发展条件明显改善。限额以上固定资产投资完成363亿元，增长49.2％。城镇建设扎实推进，统筹抓好中心城市和小城镇建设，实施了给排水、垃圾处理、集中供热等基础设施项目226个；全市房屋建设面积655万平方米，超过前两年的总和；新区基础设施条件进一步改善，年度完成投资7亿元，市民广场、公园、道路、管网、绿化等基础设施进一步完善并开工房屋建设面积70万平方米；新建了满洲里新国门、呼伦贝尔大剧院等一批城市标志性建筑，城市品位进一步提升。我市荣获“中国十大特色休闲城市”称号。成功承办全区小城镇建设现场会。交通条件明显改善，滨洲铁路海满段复线改造全面完成，两伊铁路全力推进，启动了阿荣旗—扎兰屯地方铁路项目；全区“十一五”期间最大、我市第一条高速公路绥满国道主干线阿荣旗—博克图—牙克石段开工建设；开工通乡公路18条，实施通村砂石路项目116个。海拉尔、满洲里机场达到4D级标准；开辟国内外航线19条，年进出港客流量45万人次。水利建设进程加快，呼伦湖水资源配置及水环境治理工程完成年度任务，红花尔基水利枢纽完成坝体工程建设。国家级生态市创建工作扎实推进，生态环境持续改善。加大了资源勘探和保护力度，科学利用资源、合理保护环境水平进一步提高。

――各项事业全面发展。科技支撑能力不断增强，科技创新步伐进一步加快。各级各类教育全面发展，呼伦贝尔职业技术学院开工建设，内蒙古大学满洲里学院招录本科新生。倾力打造独具呼伦贝尔民族特色的文化品牌，《五彩呼伦贝尔儿童合唱》等五张文化名片极大提升了地区影响力；《蒙古之源—呼伦贝尔历史遗迹考证》大型纪录片开机拍摄。公共卫生设施全面加强，医疗卫生条件明显改善。人口和计划生育工作得到加强，低生育水平保持稳定。全面加强了人事人才工作，人才支撑作用进一步显现。体育事业蓬勃发展，我市有6名运动员参加北京奥运会，占全区参赛运动员的一半。实施了中央广播电视无线覆盖工程，全市广播电视覆盖率分别达到90%和88.6%。精神文明建设成果丰硕，开展了道德模范评选表彰活动和征集呼伦贝尔城市精神表述语活动，举办了莫旗、鄂温克旗成立50周年庆典。满洲里荣获“全国文明城市”称号。全力支援汶川抗震救灾，累计捐款捐物5000余万元。民族、宗教、外事侨务、信息化、统计、物价、档案史志、人防、气象、地震、妇女儿童、老龄、残联、工商联、红十字会、邮政、电信等工作取得了新成绩。

——群众得到更多实惠。深入实施“六个一”幸福指数工程。社会事业和民生工程投入97.9亿元，增长59.4％，占全市财政总支出的59.1%。职工收入大幅增长，市直行政事业单位人均增加津补贴6000元、各旗市区增加5136元，森工集团在岗职工增资1600元，农垦职工增资1000元，鄂伦春猎民生产性收入首次人均超过1000元。新型农村牧区合作医疗深入推进，参合率达到93.2%。社会保障体系逐步健全完善，覆盖范围进一步扩大。深入实施整村推进扶贫到户和移民扩镇工程，解决了10万贫困人口的温饱问题。

在市二届二次人代会上，市委、市政府承诺的为民办10件实事全部兑现。一是呼伦贝尔中心城区城镇居民供水水源改造扩建工程净水厂生产、生活区主体工程整体封顶。二是新建13个城镇居民社区活动场所全部投入使用。三是市特殊教育中心主体工程完工，阿荣旗岭东特殊教育中心改扩建工程投入使用。四是对蒙语授课高中家庭困难学生进行补助，1331名学生受益。五是城镇居民基本医疗保险全面启动，参保缴费人数321976人。六是新建6处自来水工程，解决了3.5万人4.13万头只牲畜的饮水安全问题。七是发放低收入家庭廉租住房保障租赁补贴1788万元，14447户家庭受益；新建及收购廉租住房1729套，实物配租建筑面积79133平方米。八是城镇新增就业2.8万人，完成全年任务的112%；援助了160名“零就业”家庭和特困下岗失业家庭子女入学。九是帮助鄂伦春族猎民及根河市敖鲁古雅鄂温克族猎民发展生产，发放生产生活补助，1350人受益。十是为9.48万户城市低保家庭一次性发放燃煤补贴948万元。

20个公共公益项目稳步实施。海拉尔机场路改扩建和节水灌溉推广工程全部完成。呼伦贝尔结核病院住院楼、市蒙医医院和鄂伦春自治旗文化体育中心等项目完成工程主体。海拉尔火车站改造、市体育场、满洲里新国际货场一期工程、呼伦贝尔职业技术学院一期工程、草原保护和草原生态补偿、三少民族自治旗通乡公路改造、沙地治理、新区基础设施建设、满洲里棚户区改造、阿扎地方铁路、绥满国道主干线阿荣旗—博克图—牙克石高速公路、《天骄•成吉思汗》实景演出、呼伦贝尔民族文化园和牙克石凤冠高级滑雪场等跨年度公共公益项目有序推进。

――民主法制建设得到加强。按照打造高效、务实、廉洁、为民政府的要求，全面加强政府自身建设。自觉接受人大依法监督和政协民主监督，共办理人大代表议案、建议219件，政协委员提案367件。进一步加强了与各民主党派、工商联和无党派人士的联系。审计监督力度进一步加大，廉政建设和反腐败斗争不断加强。深入推进“安全稳定呼伦贝尔”创建活动，开展了党政主要领导大接访活动，群众安全感和满意度明显提高。安全生产形势稳定，事故总量下降17.5%。积极支持军队和国防后备力量建设，呼伦贝尔市和满洲里市再次被评为“全国双拥模范城”。

各位代表，过去一年，我们在解放思想、发展经济、保护生态和改善民生等领域都取得了新成效，实现了新跨越。这些成绩的取得，是自治区党委、政府和市委正确领导、几大班子团结奋进的结果，是全市各级干部励精图治、各族群众奋力拼搏的结果，是社会各界大力支持和帮助的结果。在此，我代表呼伦贝尔市人民政府，向辛勤工作的各族干部群众，向所有关心支持呼伦贝尔发展的同志们、朋友们，表示衷心的感谢和崇高的敬意！

在看到成绩的同时，我们也深刻地认识到面临的困难和问题还很多，主要体现在以下几个方面：一是受金融危机的影响，去年下半年以来，大宗工业产品价格持续走低，给企业发展和项目建设带来了诸多困难。二是国家取消边境小额贸易“双减半”优惠政策和增值税转型改革，对我市进出口贸易、财税增收影响较大。三是我市经济总量还不大，产业集群化发展水平仍很低，工业在建项目多，经济发展尚处于建设周期。四是农牧林区基础设施依然薄弱，产业化水平较低，抵御自然和市场风险能力不强，农牧民增收还存在许多不确定因素。五是非公有制经济和现代服务业发展相对滞后，经济发展活力不强。六是草原沙化扩展趋势没有得到有效遏制，沙区综合治理任务艰巨。七是林区和部分“三少”民族群众生活比较困难，广大人民群众还不富裕。八是部分干部群众思想解放程度不够，经济发展环境有待进一步优化。对于这些问题，我们要采取有力措施，认真加以解决。

二、2009年主要任务

2009年，是希望与挑战并存的一年，更是我市承前启后、跨入大发展的关键年。我们既要积极应对世界金融危机的冲击，又要牢牢把握国家扩大内需带来的新机遇；既要努力克服经济增长乏力等诸多困难，又要紧紧抓住新一轮项目建设带来的动力；既要有效化解政策调整对增收带来的巨大压力，又要积极寻求产业转移中催生的活力。总之，今年我们面对的挑战前所未有，面临的机遇也前所未有。在复杂的形势和严峻的考验面前，我们一定要看到我市经济发展基本面好于全国全区，一定要看到我市资源优势、产业组合优势也好于全国全区，一定要把困难估计得更多一些、把机遇抓得更紧一些、把工作做得更实一些，防止由于估计不足和准备不够而陷入被动，更要防止由于过度悲观而丧失发展机遇。我们坚信：只要全市上下进一步坚定发展信心，进一步解放思想，深化改革，扩大开放，在应对挑战中把握机遇，在开拓进取中争创优势，在改革发展中提高工作水平，就一定能够继续保持经济社会又好又快发展的良好势头。

今年政府工作的总体要求是：全面贯彻党的十七大、十七届三中全会、全区经济工作会议和市委二届十一次全委会精神，深入落实科学发展观，坚定不移地走生态文明建设和又好又快发展之路，以“抓机遇、扩内需、打基础、保增长”为主线，以保障和改善民生为出发点和落脚点，着力优化发展环境，着力做大经济总量，着力抓好工业园区和各类项目建设，大力推进“三化”互动和新型边境区域合作，全面加强精神文明、民主法制和政府自身建设，全力打造自治区新的经济增长极，以优异成绩迎接新中国成立60周年。

今年全市经济社会发展的预期目标是：地区生产总值增长16％，达到758亿元。限额以上固定资产投资增长28.1%，达到465亿元。财政总收入增长10％，达到110.13亿元；地方财政总收入增长10％，达到81.63亿元。城镇居民人均可支配收入增长15％，达到13914元。农牧民人均纯收入增长17％，达到5921元。城镇登记失业率控制在4.3％以内。人口自然增长率控制在5.18‰以内。全面完成自治区下达的节能减排目标任务。

为实现上述目标，今年要全力抓好十项重点工作：

（一）以扩内需、保增长为重点，全力深化改革、扩大开放。当前，国家为了应对金融危机采取了扩大内需、放宽信贷等一系列保增长的经济政策，我们一定要抓住这一历史性机遇，全力深化改革、扩大开放，切实抓好各级各类项目建设，为经济社会大发展奠定更加坚实的基础。一要进一步深化各项改革。为了更好地应对金融危机的冲击，我们必须加大各项改革力度，从体制机制上增强防范和化解金融危机的能力。重点抓好金融和财税体制改革，全面推进农村牧区综合改革和事业单位人事制度改革。建立健全现代企业制度，提高企业自我发展、自我壮大的能力。二要努力扩大开放。今年的招商引资形势不同于往年，各旗市区、园区和基地要搞好与大企业、大集团的对接，进一步加大对长三角、珠三角和环渤海经济圈等发达地区的择商选资力度，积极承接产业转移。今年要组织2—3次集中统一的大规模招商活动。全年引进国内（市外）资金372亿元，增长10%；实际利用外资6613万美元，增长15%；各旗市区要引进2—5个5000万至5亿元的中小项目。三要全力争跑项目。着力做好项目的规划和编制工作，积极争取国家和自治区在支线机场、生态建设、廉租房和棚户区改造、农牧林区基础设施、交通能源、教育卫生等方面的项目和资金，带动地方性投入，通过3—5年的努力，显著改善我市城乡发展条件。各级各部门要准确把握国家和自治区政策要求和投资重点，积极谋划储备一批符合地区经济社会发展的重大项目，市本级建立形成13大门类、总量达到1400个以上的项目库。各旗市区要同步抓好此项工作。各级各行业要加强协调、通力配合，全力向上争取，确保更多项目资金落实到呼伦贝尔。四要积极扩大消费需求。要多渠道提高城乡居民收入水平，健全社会保障体系，切实减少消费者的后顾之忧，增强消费信心。推进“万村千乡”、“农机下乡”、“家电下乡”等市场工程建设，大力开拓农村牧区消费市场。

（二）以强抓工业园区为重点，全力推进工业经济快速发展。保工业就是保增长、保稳定、保大局。要把工业园区和重点工业项目作为主攻方向，切实加强对工业经济运行的组织调度和协调，确保工业经济平稳快速发展。完成工业固定资产投资240亿元，增长11.3%；全部工业增加值达到239亿元，增长20%。一要强力推进工业园区建设。把工业园区建设作为工业经济发展的重中之重，强力加以推进。成立工业园区领导小组和办公室，统筹抓好全市园区工作。按照点状布局、集中发展的要求，高起点、全方位科学制定园区总体规划，满足长远发展的需要。实行园区带基地的发展模式，今年市本级投入3亿元政府引导性资金，通过融资和市场化运作放大到15亿元，用于建设、提升、改造各级各类园区和基地。到2012年，园区基础设施投入要达到100亿元以上，重点加强呼伦贝尔经济技术开发区、呼伦贝尔循环经济工业园区、陈旗和岭东等9大工业园区基础设施建设，全力抓好牙克石生物制药、鄂温克伊敏、新左旗嵯岗和新右旗甲乌拉等18个工业化点状基地基础设施建设。要加大招商引资和择商选资力度，着力引进一批大中型项目入园建设，今年，每个园区至少引入2—5个工业项目，园区项目工业固定资产投资达到190亿元以上，完成工业增加值180亿元以上；到2012年，园区累计工业固定资产投资达到1000亿元以上，完成工业增加值700亿元以上，吸纳就业15万人以上。各旗市区、相关部门要高度重视工业园区建设，拿出更多的财力精力，解决园区建设中存在的问题。二要强力推进重点工业项目建设。项目建设是工业经济发展的生命线。要紧紧围绕“115535”工程，全力实施88个重点工业项目。煤炭新增产能600万吨，达到5000万吨，重点抓好伊敏、宝日希勒2个超千万吨煤矿扩能和通大五牧场长焰煤基地的达产达效。新开扎尼河露天、宝日希勒二号露天、新大洲五九等煤矿项目。电力新增装机95万千瓦，达到380万千瓦，确保华能海拉尔、扎赉诺尔两个热电联产和国华、华能新源风电项目建成投产，抓好电力外送通道和360万千瓦一期外送电源建设，做好400万千瓦二期外送电源和5个旗市热电项目前期工作。化工今年要形成20万吨甲醇和为PVC配套的20万吨电石生产能力，重点抓好东能化工120万吨甲醇、金新80万吨尿素、大唐30万吨尿素、新大洲12万吨PVC等项目建设，积极抓好神华和汉能两个煤化工项目前期工作。有色金属（黑金属）冶炼新增日处理矿石4万吨，达到5万吨，加快推进乌努克吐山铜钼矿、谢尔塔拉铁锌矿、八大关铜钼矿等采选项目建设，力争开工20万吨铅锌和10万吨铜冶炼项目。石油要全力支持海塔区域的勘探和开发，推进海塔石油开发指挥部的注册工作，产量增加21万吨，达到85万吨。水泥新增产量220万吨，达到366万吨，年内争取建成蒙西阿荣旗和满洲里两个粉磨站，推进北疆扎兰屯熟料项目建设。要高度重视非资源产业的培育和发展，重点抓好“中俄电子科技园”、“苏州—呼伦贝尔电子工业园”和沈阳同联生物制药等项目建设。三要强力优化资源综合配置。加强对战略性资源的掌控，以资源的优化配置促进产业结构优化升级。加大用水结构调整和水权置换力度，以水资源为组合条件布局重大工业项目；将煤炭等优势资源向大型企业、优势产业集中配置，提高资源综合开发利用率。四要强力抓好工业经济运行调度。把存量经济作为保工业增长的重点，积极帮助企业协调和预测好市场条件、协调和疏导好地方工业产品的外运条件、协调和推动好企业的技改升级条件、协调和衔接好相关企业的产业化组合条件，充分发挥存量经济支撑作用。针对当前金融危机的影响，制定出台扶持企业应对金融危机加快发展的政策和措施，积极向上争取扶持资金，重点落实好国家和自治区扶持企业发展的各项政策，帮助企业开拓市场、解决流动资金贷款等突出问题。五要强力落实节能减排任务。积极扶持生态节能型产业优先发展，抓好煤炭、电力、冶金和建材等重点行业节能减排工作，全面完成自治区下达的节能减排任务。

（三）以促进农牧民增收为重点，全力推进现代农牧业建设。贯彻落实党的十七届三中全会精神，用足用好国家扩大内需的各项政策，加快推进农牧林区基础设施建设，全面促进现代农牧业发展。第一产业增加值达到162亿元，增长7%。粮食总产量力争达到100亿斤。牲畜存栏总数稳定在1500万头只。一要大力提升农牧业产业化水平。把提升农牧业产业化水平作为促进农牧业发展、农牧民增收的主渠道，因地制宜，全面推进。积极扶持农畜产品加工企业发展壮大，支持乳、肉、薯、粮油等龙头企业进行扩能改造，全面提高市场竞争力和辐射带动力。加快原料基地建设，按照农牧业产业化生产要求，建设乳、肉、粮油、马铃薯、饲草饲料、木材和特色养殖业等7大产业基地，启动实施粮食增产工程，重点抓好高油大豆、专用玉米、马铃薯、专用小麦大麦、水稻、杂粮杂豆等6大项目区建设，确保龙头企业的原料需求。鼓励龙头企业建设稳固的原材料生产基地，把企业经营向一产延伸。抓好农牧业适用技术的推广和应用，加强农畜产品质量安全检验检测。大力发展专业合作社，提高农牧业生产的社会化和组织化程度。二要扎实推进新农牧林区建设。继续推进16个新农牧林区建设试点的样板村嘎查建设。紧紧抓住国家加大民生工程投入的机遇，加强农牧林区基础设施建设，积极争取农田基本建设、交通、卫生、教育、危房改造等方面的建设项目。实施“百村美化”工程，大力整治农村环境“脏、乱、差”的状况。围绕七大主导产业和10个绿色品牌，引导扶持村嘎查培育1—2个支柱产业，为新农牧林区建设提供产业支撑。三要全面落实各项强农惠农政策。认真执行提高粮食最低收购价政策，不断完善粮食直补、良种补贴、农机具购置补贴、农业生产资料综合补贴等各项农牧业补贴制度。稳定和完善农村牧区基本经营权制度，鼓励和支持土地、草牧场承包经营权有序流转。全面推进集体林权改革，进一步明晰集体林地的使用权和林木所有权。加快推进村镇银行、资金互助社等新型农村牧区金融机构试点工作，规范和引导民间借贷行为。大力推进农牧业保险体系建设，建立风险分散机制。针对当前鲜奶价格走低等问题，各级政府要及时启动价格调节基金，采取帮助企业协调贷款、开拓市场等措施，使企业度过难关；同时，要督促企业提高鲜奶收购价格，保护奶农的利益。四要多渠道促进农牧民增收。加大财政投入，确保各级财政对农牧林业投入增长幅度高于经常性收入增长幅度。加快农牧林区二、三产业发展，提高管理水平，搞好市场衔接，切实增加农牧民经营性收入。积极支持森工集团深化改革，推进林区产业化发展，加快区域经济一体化进程。加大农牧民和林区富余劳动力转移力度，拓展农牧民和林区职工增收渠道，农牧民转移培训6000人，转移就业6万人，非农牧林收入比重力争超过20%。

（四）以旅贸业发展为重点，全力繁荣第三产业。旅游重点抓景区，培育知名品牌，提高旅游承载力。全年接待游客超过630万人次，增长15%以上；旅游总收入达到108.75亿元，增长25%。重点景区建设要以打造在国内外知名的旅游景区为目标，全力抓好呼伦贝尔民族文化园二期、《天骄•成吉思汗》实景演出、世界反法西斯战争海拉尔纪念园、满洲里新国门、牙克石凤冠高级滑雪场、柴河、诺门罕战争遗址、敖鲁古雅、嘎仙洞、宝东山祭祀、巴彦胡硕、莫尔道嘎森林公园、达斡尔民族园、东北抗联英雄纪念园等重点景区建设。景区建设要高起点、高立意、大投入，全面体现民俗、文化和历史内涵。景区管理要高标准、严要求，全面提高接待水平。旅游精品线路要围绕重点景区，突出自然生态、历史文化、异域边境，积极打造“五彩呼伦贝尔民俗体验主题游”精品线路。旅游节庆活动要继续推动四季旅游均衡发展，以红春杜鹃节、绿夏草原那达慕、金秋大兴安岭森林摄影节、银冬冰雪那达慕等四季旅游品牌为载体，精心打造富有地方民族文化特色的旅游节庆活动。旅游宣传促销要借助中央电视台和旅游卫视等平台，全面开展促销活动，不断提高我市美誉度和对国内外游客的吸引力。要重点加强旅游大通道建设，国内航线今年要突破20条，国际航线力争开通俄罗斯、日本、蒙古包机。外贸要深入推进以满洲里为龙头，以沿边口岸旗市为两翼的新型边境区域合作，全力做大做强满洲里，外贸进出口总额完成28.8亿美元，增长3%，进出口货运量完成2555万吨，增长5%。一要强力推进出口基地建设。针对俄蒙市场需求，进一步加强以服装、电子、农畜产品加工和木制品加工等产品为主，沿301国道和滨州铁路沿线的出口基地建设。二要强力推进境外资源开发。全力做好大庆石油塔木察格油田、诺永达拉果铅锌矿、乌兰铅锌矿、别列佐夫铁矿、森工集团境外木材加工等境外项目的跟踪服务，促进企业加快发展。三要强力推进口岸基础设施建设。进一步完善口岸联检大楼、满洲里国际货场等基础设施建设。加快制定黑山头、额布都格电子口岸发展规划和实施方案。制定完善阿日哈沙特、室韦、海拉尔航空口岸发展总体规划。积极做好额尔古纳、新右旗3条跨境铁路立项后的推进工作，不断提升口岸经济发展能力。金融要把促进经济平稳较快发展作为首要任务，进一步优化金融生态环境，提高担保能力，缩小存贷比例，扩大信贷规模。要调动国有商业银行、农村信用合作社的积极性，着力引进股份制商业银行，发展小额贷款公司和村镇银行，为经济社会发展提供坚强的金融保障。物流要加快以海满牙扎为重点的物流市场体系建设，促进现代服务业快速发展。

（五）以提供优良服务为重点，全力扶持非公有制经济发展。大力优化发展环境，要让非公有制经济“唱主角、坐正席、挑大梁”，全力壮大非公有制经济。非公有制经济占全市经济比重要达到45.5%。一要壮规模。认真落实扶持非公有制经济发展的各项政策，既要高度重视引进大型非公企业入驻，更要加速培育本地知名非公企业。进一步拓宽非公企业融资渠道，充分发挥中小企业投资担保公司的作用，壮大担保实力，放大担保规模，搞好银企项目对接，为非公企业发展提供信贷支持。引导非公企业参与矿产资源开发加工、进出口产品加工和新农牧林区建设，引导非公企业向园区和基地集中，为非公企业发展壮大创造更为广阔的发展空间。二要提层次。实施好中小企业促进工程，努力培育非公有制经济产业集群，进一步提高非公有制经济的集约化和规模化水平。大力支持非公企业与相关科研机构联合，实现科技成果和人力资源共享。非公企业要与入驻我市的大集团、大企业搞好产业配套，借助大企业在科研、资金等方面的实力，实现产业升级。三要增后劲。进一步研究出台扶持非公有制经济发展的政策措施，加强非公经济人才的引进、开发和培养，切实提高非公经济人才队伍整体素质。重奖业绩突出的非公企业家，在全社会形成关心重视非公经济发展的良好氛围。

（六）以呼伦贝尔沙区综合治理为重点，全力推进生态保护与建设。把呼伦贝尔沙区综合治理作为今年全市公共公益项目的一号工程，动员全社会力量，遏制沙化的蔓延。要加大资金投入，年内要投入资金1.5亿元，治理沙化面积100万亩，其中，人工造林35万亩，封沙育林（草）65万亩。要优化治理布局，制定沙区综合治理规划，以“五点、三带、两区”为重点，对危及交通运输、人民生产生活安全的区域进行重点治理。要建立补偿机制，促进生态保护与经济社会协调发展。要科学治沙，采取人工治理与自然修复相结合的综合措施，继续推进“退、封、建”三项生态治理工程。稳步实施生态移民，逐步把牧民从生态脆弱区有序转移出来。要强化后期管护，制定出台责权利相统一的后期管护长效机制，做到治理一片、管好一片、守住一片，巩固沙区治理成果。要发挥好大企业的作用，组织动员10—20户资源开发型企业参与沙区综合治理，增强全社会保护生态环境的使命感和责任感。要积极发展沙产业，支持生态建设产业化发展，引进龙头企业进行加工转化，促进沙区治理与发展生态经济同步推进。以创建国家级生态市为目标，全面加强生态建设与保护。在加大治沙力度的同时，继续加大大兴安岭生态建设与保护力度，加大农区生态建设与保护力度，加大草原建设与保护力度，重点抓好天保工程、重点公益林保护、三北四期防护林建设等重点工程，统筹推进退耕和禁牧休牧工作，继续退耕还林还草100万亩，草地围栏400万亩，休牧5000万亩，禁牧500万亩。认真执行环境影响评价和“三同时”制度，严把建设项目审批核准关。加大项目建设的执法监察和重点区域、流域及重点污染源的治理力度，确保不发生新的环境污染。

（七）以加强基础设施建设为重点，全力改善城乡发展条件。城镇重点抓棚户区，把实施安居工程作为今年最大的实事，实施廉租房、棚户区改造和牧区生态移民工程，建设面积不少于100万平方米。重点城市建设要突出特色打造，海拉尔以打造呼伦贝尔经济发展核心极为目标，倾力建设民族风情魅力城市，重点抓好火车站站前环境综合整治和“城中村”改造，建成哈萨尔斜拉桥、呼伦贝尔古城等标志性建筑；满洲里要突出欧式古典建筑艺术特色，重点抓好口岸世界城双子座、欧式旅游观光婚礼宫等十大标志性建筑。城镇建设要以完善城镇基础设施为重点，投资41.8亿元，实施20万元以上项目229个，重点抓好污水处理、垃圾处理、绿化、城镇道路的建设和改造。加强小城镇建设与管理，切实提高承载力，构筑层次分明、特色鲜明的小城镇体系。加快新区建设步伐，完善商业、教育、卫生等配套设施，大力推进市直在建单位入驻；启动新区河东部分建设。公路计划投资52亿元，拉布达林—黑山头、根河—满归两条公路要建成通车，绥满国道主干线阿荣旗—博克图—牙克石高速公路完成年度建设任务，争取开工建设海拉尔—阿木古郎、阿木古郎—杜拉尔桥一级公路，积极做好牙克石—海拉尔高速公路和满洲里—阿拉坦额莫勒、阿拉坦额莫勒—阿木古郎一级公路前期工作。抓住我市列入全国14个农村公路示范工程单位的机遇，全面推进乌尔其汗—宜里六连等19条通乡、通村公路建设。铁路要确保两伊铁路建成通车，扎实推进阿荣旗—扎兰屯地方铁路建设，做好乔巴山—阿日哈沙特—伊尔施、室韦—莫尔道嘎、海拉尔—黑山头和满洲里—乔巴山等项目前期工作。民航要重点开展海拉尔机场改造前期工作，完成满洲里机场候机楼改扩建二期工程，加快推进根河、鄂伦春、莫尔道嘎和扎兰屯支线机场前期工作。水利建设要围绕经济社会发展筹划一批新的水利工程，重点完成第三批10座病险水库除险加固建设任务和呼伦湖水资源配置及水环境治理工程，继续抓好红花尔基水利枢纽工程建设，加快中小河流治理工程进程，确保尼尔基水利枢纽下游内蒙古灌区等项目前期工作取得突破性进展。

（八）以促进基本公共服务均等化为重点，全力推动社会事业发展。全面加强科技工作，大力提高科技对经济发展的贡献率。推动各级各类教育健康发展，加快呼伦贝尔职业技术学院建设，完成市特殊教育中心搬迁，全面开展呼伦贝尔学院本科教学迎评促建工作。加强医疗卫生工作，进一步完善农村牧区卫生服务体系，抓好重点疾病预防控制；积极推行新型农村牧区合作医疗，参合率达到95%；深入开展食品、药品安全专项整治，加强对生产流通全过程监管。做好人口和计生工作，稳定低生育水平。加强老龄人口和妇女儿童权益保障，做好残疾人康复、保障和“全国无障碍城市”创建工作。发展文化、广播、电视、体育事业，继续打造呼伦贝尔五张文化名片，实施较少民族文化遗产保护工程，建设游牧文化和驯鹿文化保护区，创建呼伦贝尔博物馆城。积极申办国家级、世界级冬季体育赛事，主动争办世界儿童合唱节。加强就业培训，力争实现新增就业2.4万人，动态实现每个有就业能力和愿望的“零就业”家庭至少1人就业。积极做好社会保险政策完善和扩面征缴工作，进一步扩大农村牧区社会养老保险试点范围，继续做好城镇居民基本医疗保险工作。强化劳动监察执法，严格落实最低工资标准，维护劳动者与企业合法权益。稳步提高城乡低保标准和补助水平，抓好贫困家庭大学生的救助工作。大力实施人才强市战略，推进人才资源整体开发。全面加强财税工作，确保财税收入稳定快速增长。全面落实民族宗教政策，大力发展民族经济。认真做好统计、审计、档案史志、扶贫、人防、气象、地震、电信、质监、邮政、保险和对外联络等工作。

（九）以保障和改善民生为重点，继续实施办实事和公共公益项目。市委、市政府继续把实施“六个一”幸福指数工程作为改善民生的统领，强力加以推进。今年要继续推进2008年未完成的11个公共公益项目，其中，海拉尔火车站项目要启动1.2万平方米新站房及基础站台、无柱雨棚、路网改造等工程，同时完成站前广场及4条配套市政道路和相关设施建设。市中心城区水源地改造，今年完成净水厂工程以及供水主管线和9眼水源辐射井建设，确保2010年6月份让市中心城区居民喝上放心水、安全水。市体育场计划完成投资2亿元，年内完成土建主体工程，确保2010年竣工投入使用。呼伦贝尔职业技术学院年内要完成主教学楼、学生宿舍楼和实习基地等项目建设。呼伦贝尔民族文化园要继续推进一期工程建设，抓好北山舍利塔内部配套、塔周边环境治理和中华天竺园的八大圣景建设；二期工程要重点抓好园区总体规划，启动蒙古、达斡尔、鄂温克、鄂伦春、俄罗斯五个民族文化景区工程。《天骄•成吉思汗》实景演出要抓好剧目编排、设备入场等基础性工作，完成水电路等设施建设和周边环境整治，做好看台建设和演员招募工作，确保6月下旬试演。阿荣旗—博克图—牙克石高速公路年内确保完成投资38亿元，重点抓好路基、桥涵、隧道和路面备料等工程。满洲里新国际货场一期要完成投资11亿元，完成铁路国际货场主体工程，建成汽车、煤炭散料和化学危险品专业货场。牙克石凤冠高级滑雪场要完成全部工程。全面开工建设图书馆和档案馆库。继续抓好城镇低保和“零就业”家庭子女助学、城市低保家庭燃煤补贴等5件2008年确定的实事。

根据自治区确定的2009年为民办实事和我市所确定的为民办实事项目，多渠道筹措资金8.5亿元，再为民办20件实事。其中：承办自治区的实事10件：一是提高11万城镇居民低保标准。二是提高农牧民低保补助标准，惠及9.5万人。三是企业退休人员人均养老金标准提高11%以上。四是使城镇居民基本医疗保险参保人数达到50万人以上。五是提高农牧民的新型农村牧区合作医疗补助标准，惠及71.2万人。六是对困难家庭大中专学生进行资助，惠及21622人。七是实行农牧业保险补贴，惠及17万农牧户。八是解决17.5万人饮水安全问题。九是新建廉租住房9365户43.59万平方米，占应保尽保户数的42.6%。十是取消部分地方制定的收费项目，为企业和群众减轻负担423万元。我市为民办实事10件：一是全面启动安居工程，实施廉租房、棚户区改造和牧区生态移民工程，建设总面积不少于100万平方米，惠及15万人。二是实施呼伦贝尔孤老残幼福利家园项目，建设儿童福利院、孤老院及附属生活区、休闲区，改善弱势群体的生活条件。三是启动少数民族传统文化保护开发工程，挖掘民族文化内涵，传承民族文化精神，打造民族文化品牌。四是实现蒙语广播节目全覆盖，为1.2万户牧民家庭配发收听器材。五是设立全市道德模范奖励助困基金，重奖道德楷模，扶助道德模范，在全社会弘扬文明道德风尚。六是改善城镇公交状况，新增10条公交线路及168个设施，解决群众出行难问题。七是为全市80所农村牧区寄宿制学校改善生活设施，惠及学生1.5万人。八是对患先天性心脏病的贫困儿童进行康复手术补贴。九是为全市独生子女、农牧区双女结扎户女孩和计生干部交纳人身意外伤害保险，惠及7.6万人。十是提高“五保户”供养标准，惠及5316户家庭。

根据扩内需、惠民生、保增长的需要，今年再新启动10个公共公益项目。一是沙地综合治理工程，治理沙地100万亩，涉及新左旗、新右旗、陈旗、鄂温克旗、海拉尔区和满洲里市6个旗市区。二是工业园区建设项目，做大做强9个工业园区和18个产业化基地。三是中心城新区河东基础设施建设工程，完善新区基础设施条件。四是全市各旗市区污水、垃圾处理项目全部开工。五是中心城区主街道综合治理工程，改善城市发展条件。六是新建中心城新区中小学，完善新区功能。七是巴尔虎草原重点景区建设项目，打造天堂草原代表性景区。八是海拉尔北出口道路升级改造工程，全部实现中心城区出口道路升级改造目标。九是实施民兵预备役基础设施改造工程，巩固双拥建设成果。十是农村牧区文化卫生建设工程，新建15个乡镇综合文化站和39个村卫生室。

（十）以打造平安稳定呼伦贝尔为重点，全面加强精神文明和民主法制建设。全面推进文明城市、乡镇和社区等群众性精神文明创建活动，深入开展道德模范学习宣传活动，全面提高公民的思想道德素质和全社会的文明程度。深入开展全国双拥模范城创建工作，进一步加强国防教育和国防后备力量建设。自觉接受人大的依法监督和政协的民主监督，认真办理人大代表议案、建议和政协委员提案、建议。加强新闻媒体舆论监督，广泛听取各民主党派、无党派人士和专家学者的意见。支持工会、共青团、妇联等人民团体的工作。推进政务、村务和厂务公开，不断扩大基层民主。高度重视群众来信来访，妥善处理信访突出问题及群体性事件，认真解决群众反映强烈的热点、难点问题。继续开展“安全稳定呼伦贝尔”创建活动，全面加强社会治安综合治理。认真落实各项安全生产责任制，切实抓好煤矿、交通、森林草原防火和高危行业的安全生产，确保人民群众的生命财产安全。积极建立完善群体性事件预警、应急和处置机制，提高保障公共安全和处置突发事件的能力。

各位代表，今年是呼伦贝尔在打造自治区新的经济增长极进程中最为关键的一年，市委、市政府决定将今年确定为“优化发展环境年”。我们必须切实优化发展环境、加强政府自身建设，为经济社会平稳快速发展提供坚强保障。良好的发展环境是招商引资、择商选资的助推器，是项目建设的生命线，是加快我市大发展的根本保障。大力精减审批事项，减少办事环节，进一步加强行政审批中心建设。各部门要切实增强勤政廉政意识，强化服务功能，提高办事效率，正确行使权力，为企业、为基层做好综合服务，要在全市开展“百企评部门、评行风”活动。要将优化发展环境与学习实践科学发展观活动紧密结合起来，全面加强政府自身建设，打造人民满意的政府。要大力提高政府的执行力，全面加强队伍建设，严明奖惩措施，强化工作落实，确保政令畅通；要大力提高政府的创新力，更新思想观念，创新工作机制，完善工作方法，创造性地开展各项工作；要大力提高政府的凝聚力，加强团结协作，高度关注民生，大力激发各级干部群众创业热情，形成风清气正、一心一意谋发展的局面；要大力提高政府的公信力，坚持依法行政、廉洁从政，确保权力在阳光下运行，树立良好的公众形象。

各位代表，今年各项工作任务十分繁重，让我们在市委的坚强领导下，努力化解金融危机带来的不利影响，紧紧抓住发展这个中心任务不动摇，团结带领全市270万各族人民，解放思想、振奋精神、抢抓机遇、攻坚克难，为打造自治区新的经济增长极、实现呼伦贝尔全面振兴而努力奋斗！
